# Small RNA AvrA Regulates IscR to Increase the Stress Tolerances in SmpB Deficiency of *Aeromonas veronii*

**DOI:** 10.3389/fcimb.2019.00142

**Published:** 2019-05-08

**Authors:** Dan Wang, Hong Li, Xiang Ma, Yanqiong Tang, Hongqian Tang, Xinwen Hu, Zhu Liu

**Affiliations:** Key Laboratory of Tropical Biological Resources of Ministry of Education, School of Life and Pharmaceutical Sciences, Hainan University, Haikou, China

**Keywords:** oxidative, stress, iron deficiency, iron/sulfur clusters, virulence

## Abstract

The superbacteria *Aeromonas veronii* displays not only a strong pathogenicity but also the resistance to nine kinds of antibiotics, resulting in the economic losses and health hazards. Small Protein B (SmpB) plays an important role in protein quality control, virulence, and stress reactions. Transcriptomic data revealed that expressions of the type IV pilus assembly and type VI secretion system (T6SS) proteins were downregulated in SmpB deficiency, indicating that the virulence of *A. veronii* might be attenuated. Although SmpB deletion decreased colonization in the mouse spleen and liver, LD50 of the *smpB* mutant was not altered as expected, compared with the wild type. Further, the transcriptomic and quantitative RT-PCR analyses showed that the combination of the downregulated AvrA and the upregulated iron-sulfur protein activator IscR, mediated the oxidative tolerance in *smpB* deletion. Next a reporter plasmid was constructed in which the promoter of *iscR* was applied to control the expression of the enhanced green fluorescent protein (eGFP) gene. When the reporter plasmid was co-expressed with the AvrA expression into *E. coli*, the relative fluorescence intensity was decreased significantly, suggesting that AvrA bound to *iscR* mRNA by base pairing, which in turn relieved the inhibition of *iscR* and intensified the downstream iron-sulfur proteins. Collectively, the *smpB* mutant exhibited an attenuated virulence in mice and enhanced tolerances to oxidative stress. This study demonstrates the complexity of gene regulation networks mediated by sRNA in systems biology, and also reflects the strong adaptability of superbacteria *A. veronii* in the process of evolution.

## Introduction

With the application of antimicrobial drugs, a wide range of mechanisms have evolved for bacteria to combat the huge selection pressures of antibacterial agents. Superbacteria have accumulated in a long evolutionary history, while the abuse of antibiotics has accelerated its formation. The superbacterium *Aeromonas veronii* (*A. veronii*) has been isolated, which has displayed not only a strong pathogenicity but also the resistance to nine kinds of antibiotics (Liu et al., 2016, 2018). As *A. veronii* infects fish, it causes fish canker and perforated disease, eventually resulting in economic losses. Furthermore, *A. veronii* increases human diseases such as neonatal sepsis, diarrhea and dysentery (Aguilera-Arreola et al., 2007). In view of the wide array of infections to the host and a strong tolerance to antibiotics, *A. veronii* is worth researching further, as a model strain.

Proteins are the material basis of life. If there is a mistake in the protein synthesis process, ribosome rescue should occur in order to maintain life. Trans-translation which is composed of small protein B(SmpB) and transfer-message RNA (tmRNA) is the predominant form of ribosome rescue (Himeno et al., 2014). The malfunction of trans-translation exhibits more sensitivity to virulence and tolerance in most pathogens such as *Mycobacterium tuberculosis, Legionella pneumophila*, and *Neisseria gonorrhoeae* (Keiler, 2008, 2015). However, the alternative pathways have evolved to compensate for the deficiency of trans-translation. For example, the alternative rescue factor A (ArfA) acts to release the stocked mRNA in Proteobacteria and mycobacteria, and the alternative rescue factor B (ArfB) appears in 34% of bacteria (Kurita et al., 2014; Huter et al., 2017; Fiedler et al., 2018). Therefore, superbacteria might have alternate ways for SmpB defects, while no reports indicate that other functions of SmpB are maintained by small RNA (sRNA).

In this study, the transcriptome analysis was compared between wild type and *smpB* knockout strains, showing that the expressions of the pilus and virulence protein were downregulated after *smpB* deletion. However, the capacities of oxidative stress and iron were increased by sRNA downregulation in *smpB* knockout. Non-coding sRNA modulates bacterial metabolism and stress by base pairing with target genes and reacts faster than transcription factor regulation (Durand et al., 2015; Oliva et al., 2015). Here a sRNA designated as AvrA was revealed to negatively regulate the expression of IscR (iron-sulfur cluster assembly transcriptional regulator) IscR governs iron hemostasis as a global regulator during growth and stress responses (Aguilera-Arreola et al., 2007), which exhibits a resistance to oxidants (Mandin et al., 2016), and represses iron-sulfur metabolism under iron starvation (Haines et al., 2015; Carrier et al., 2017). The overproduction of IscR favored the tolerance under the nutrient deficiency and oxidative, iron-limited and membrane stresses when *smpB* was knocked out and followed by AvrA downregulation.

Survival under detrimental stresses is preliminary when pathogens are infected to the host and escape from macrophage recognition in the immune system (Mandin et al., 2016). And then the bacteria are transported to tissues using blood and lymph as channels, eventually causing tissue damage. Iron metabolism is of vital importance to bacteria. The proteins composed of iron/sulfur clusters participate in multiple cellular processes, such as gene expression, DNA repair, RNA modification, central metabolism and respiration (Roche et al., 2013). Iron sulfur proteins also relate to oxidoreductase which is involved in the basic metabolism of bacteria (Xu and Moller, 2008; Ilbert and Bonnefoy, 2013). So, overexpression of IscR is vital for bacteria to survive in adverse conditions.

In summary, SmpB plays an important role in protein quality control, virulence, gene transcription, and antibiotic and stress reaction (Liang and Deutscher, 2010; Li et al., 2013; Mu et al., 2013; Mraheil et al., 2017). Unlike *Salmonella Typhimurium*, the virulence of the *A. veronii* Δ*smpB* strain did not become avirulent but maintained a strong pathogenicity. Loss of function caused by smpB deletion can be compensated through different pathways. For instance, some bacteria employ ArfA and ArfB to rescue stalled ribosomes when *smpB* is deficient (Keiler, 2015; Liu et al., 2016; Huter et al., 2017). Additionally, sRNA AvrA releases the trapped IscR promotor to activate the expression of iron sulfur proteins, remedying the iron deficiency after *smpB* destruction. In the evolutionary history of bacteria, it offers additional evidence for the formation of superbacteria.

## Materials and Methods

### Plasmids

The plasmids and primers are listed in Tables 1, 2, respectively.

**Table 1:** Strains and vector used in this paper.

**Strains or plasmids**	**Traits**	**Sources**
*E. coli* DH5α	Gene cloning strain	This lab
*E. coli* Felden	Gene cloning strain	This lab (Liu et al., 2018)
*E. coli* WM3064	Gene cloning strain	This lab (Liu et al., 2018)
*Aeromonas veronii*	Wild type strain	This lab (Liu et al., 2018)
*smpB* mutant	*smpB* deletion mutant	This lab
*smpB* complement	*smpB* complement strain	This lab
*avrA* mutant	*avrA* deletion mutant	This paper
*avrA* complement	*avrA* complement	This paper
pUC19	Report vector	This lab (Liu et al., 2016)
PRE112	Gene cloning vector	This lab (Liu et al., 2016)
PBBR1MCS-2	Gene cloning vector	This lab (Liu et al., 2018)
pDH114	Report vector	This lab (Liu et al., 2016)
pRE112-*avrA*del	avrA deletion vector	This paper
pBBR-*avrA*	avrA expression pBBR1MCS-2 vector	This paper
p*avrA*	avrA expression pUC19 vector	This paper
pavrAmutP1	P1 site mutation on p*avrA*	This paper
Φ (piscR-eGFP)	fusion vector including the promoter of iscR and eGFP	This paper
Φ (piscR mutP1-eGFP)	P1 site mutation on Φ(piscR-eGFP)	This paper
Φ (piscRmutP2-eGFP)	P2 site mutation on Φ(piscR-eGFP)	This paper

**Table 2:** Primers used in vector construction and real-time qPCR.

**Names of primers**	**Sequences (5^**′**^−3^**′**^)**	**Usage**
SP 1_F	ATGGTCGCAGAGCTTGTC	Strain validation
SP 1_R	CAGCACAATAGAACACCAGAC	Strain validation
*avrA*_F1	TCTTAGATCCAGGTACCTGGTCCAGCAACAGATCTCCGAT	avrA knockout
avrA_R1	ATTATTGTCCATTGCATTGGCCGGAGTGTTAATCATGGTG	avrA knockout
avrA_F2	ATTCCTGTCCATTGCATTGGTTGTATGTCGCCCCTAAAGG	avrA knockout
avrA_R2	CTGCAGAACCAGAGCTCTGGAGCGGGACAAACTGTTCG	avrA knockout
avrA_F0	TCTCCAGGCAAATACGCTCG	avrA knockout
avrA_R0	GAGTTCAGGCTGGTCGACTG	avrA knockout
mtarP1:	CGAGATGGATGGGGTAGACGCAGACAACATGCATCCACTTGCGG	Site mutation of p1
mtarP1cr	CCGCAAGTGGATGCATGTTGTCTGCGTCTACCCCATCCATCTCG	Site mutation of p1
mtarP2:	GACGGTCTCAACATGCATCCAGAACGCGATGATCCGCTCCCGATCGC	Site mutation of p2
mtarP2cr:	GCGATCGGGAGCGGATCATCGCGTTCTGGATGCATGTTGAGACCGTC	Site mutation of p2
11665F	GGTCTTGCCTACGTGCTTGA	Real-time PCR
11665R	CACACTCGCCTTTGGCATTG	Real-time PCR
11670F	AGCTGCAATTGAAGATCAGCG	Real-time PCR
11670R	TGTGTTCTTGATGCCAGCCG	Real-time PCR
11675F	AACGTGTTCCGGGTAACCTC	Real-time PCR
11675R	CGACCGATGGAGTCACGAAT	Real-time PCR
11680F	CGCAAACATGGTCTGGTGAG	Real-time PCR
11680R	CGGCCAAGGTAATATCCCCG	Real-time PCR
pro*iscR*_F	ACGCGTCGACGCGGTATTCTGACCTCGGTG	Fusion vector
pro*iscR*_R	CTCGCCCTTGCTCACCATCAGTCTCATGTGCCTTACCG	Fusion vector
eGFP_F	CGGTAAGGCACATGAGACTGATGGTGAGCAAGGGCGAG	Fusion vector
eGFP_R	TCGCGGATCCTTACTTGTACAGCTCGTCCA	Fusion vector
pUC19_F	AATGCAGCTGGCACGACAGG	Fusion vector
pUC19_R	CCATTCAGGCTGCGCAACTG	Fusion vector
pro*avrA*_F	CGGGGTACCCAGTCGTTGCTCCATGGCGG	Fusion vector
pro*avrA*_R	CCGGAATCCGGTCGCCAACTTCTACATCT	Fusion vector
pro*avrA* comF	CGGGGTACCAAACAGTAGCCAGGGACCGAG	Fusion vector
pro*avrA* comR	GGCCGGAATTCGCCTTTATCGCCGATCTGC	Fusion vector
pBBR1MCS-2 F	GGCACCCCAGGCTTTACACT	Complement plasmid validation
pBBR1MCS-2 R	GATGTGCTGCAAGGCGATTAAG	Complement plasmid validation

For the construction of plasmid p*iscR*-eGFP, the 200 bp-DNA fragments containing the promoter of *iscR* was amplified from the genomic DNA of *A. veronii* with primers pro*iscR*_F/pro*iscR*_R. In the meantime, the 750 bp-encoding region of eGFP was amplified from a plasmid pDH114 with primer eGFP_F/eGFP_R. The fusion was produced using the above two fragments as the templates by overlapping the PCR with primers F_PiscR/eGFP_R and by insertion into the backbone of the plasmid pUC19 to construct the Φ(p*iscR*-eGFP) vector. The plasmid p*avrA* was constructed in a similar manner using the primers pro*avrA*_F and pro*avrA*_R. To construct the gene knockout strain of Δ*avrA*, the flanking sequences of Δ*avrA* were amplified and inserted into the plasmid pRE112, resulting in the gene knockout vector pRE112-*avrA*del. The complementary plasmid pBBR-*avrA* was applied to express the extra AvrA using plasmid pBBR1MCS-2 as the backbone.

### Strains and Culture Conditions

The strains used are listed in Table 1. The derivative *A. veronii* strains were picked from frozen stocks and cultured at 30°C in Luria-Bertani (LB) broth or M9 medium with aeration. The Antibiotics included 50 μg/mL ampicillin, 25 μg/mL chloramphenicol, 50 μg/mL kanamycin. The *E. Coli* Felden strain was chosen for the expression of eGFP and sRNA (Liu et al., 2015). *E. Coli* WM3064 was selected for gene knockout by homologous recombination, which is capable of growth at 37°C in LB broth with 0.3 mM diaminopimelic acid (DAP).

The plasmid pRE112-*avrA*KO was transformed into the competent *E. coli* WM3064 and delivered into *A. veronii* by conjugation (Liu et al., 2016). The *A. veronii* Δ*avrA* was screened on LB agar supplemented with 6% sucrose and ampicillin and was further checked by sequencing.

The plasmid pBBR-*avrA* was transformed into the competent *E. coli* WM3064, and mobilized into the Δ*avrA* strain by conjugation, resulting in complementary *A. veronii* Δ*avrA*::*avrA*.

### RNA Sequencing and Bioinformatics Analysis

Strains were grown in a 10 mL M9 medium containing 50 μg/mL ampicillin at 30°C, 150 rpm for 20 h. The cells were collected and lysed. RNA samples were extracted by phenol-chloroform. The concentration and quality of RNA was detected by the Agilent 2100 Bio analyzer. The samples were treated with DNase I to eliminate double-stranded and single-stranded DNA, followed by the depletion of rRNA with the Ribo-Zero Magnetic Kit. First-strand cDNA was generated using a random primer reverse transcription, followed by a second-strand cDNA synthesis. The end of the synthesized cDNA was subjected to repair and adenylate. The adapters were ligated to the ends of these 3′-adenylated cDNA fragments. The cDNA fragments were enriched by PCR amplification with a PCR Primer Cocktail, and the purified products were sequenced on the Hiseq Xten (Illumina, San Diego, CA, USA) using the gel-free protocol. Libraries were sequenced as 100 bp paired-end reads to a minimum target depth of 2G clean data per sample. The software HISAT (V2.0.1-beta) was used for the reference genome alignment, and CPC (coding potential calculator) was used to characterize the translation possibility of new transcripts. To identify the differential expression between wild type and Δ*smpB*, Bowtie 2(v2.2.5) was used to analyze the mRNA expression. Fragments per kilobase of transcript per million fragments sequenced (FPKM) were used to normalize the gene expression levels. For each gene, the *p*-value was evaluated, and Benjamini-Hochberg false discovery rate (FDR) was applied for the correction. The differential expressions of the transcripts were estimated by the virtue of ≥1 absolute log2 -fold change and < 0.001 FDR adjusted *p*-value. These results were sorted by GO categories using Perl scripts. The GO and KEGG pathway enrichments of differential uni-genes were analyzed. The hypergeometric test was used to compare differential expression genes in the pathway with the entire genome background. Pathways with a *p* ≤ 0.05 were considered as significant. GEO accession number GSE120603.

### LD50 Test in Mice

The derivative of *A. veronii* was streaked onto M9 medium and grown for 18–20 h at 30°C. The cells were re-suspended in sterile PBS. ICR mice (male, 6–8 weeks old) were infected by intraperitoneal injection with 10^3^–10^7^ CFU/g of each strain (*n* = 8) and monitored daily for weight loss and signs of illness. The animals were euthanized if they accorded with alternate endpoint criteria including the loss of mobility. The 50% lethal dose was calculated by linear regression (Torii et al., 2015).

### Organ Coefficient

The coefficient of organs was calculated from the ratio of organ weight and body weight of the same mouse. The change of coefficient was used to characterize the degree of injury by contrasting the pathological with the normal tissue (Zhang et al., 2014).

All animal experiments were approved by the Committee of the Ethics on Animal Care and Experiments at Hainan University, and were carried out in accordance with the university guidelines.

### Measurement of Growth Curve

The condition of iron deficiency was equipped with LB medium containing 200 mM dipyridyl. M9 medium was considered as the nutrient limited condition. The strains were cultured at logarithmic phase, and then inoculated into 50 ml media at an initial OD_600_ of 0.05. The ODs were monitored with a spectrophotometer (INESA 970CRT, Shanghai, China) at regular intervals.

### Survival Under Oxidative Stress

The derivative of *A. veronii* was grown until to the OD_600_ value of 0.6 in the LB broth appended with 200 mM dipyridyl and subsequently challenged with 0–10 mM H_2_O_2_. The cultures were continuously incubated at 30°C with shaking for 10 min, and the survival colonies were counted on the LB agar plate with 50 μg/mL ampicillin.

### Real-Time PCR Experiment

The wild type and mutant strains of *A. veronii* were grown in LB and M9 supplemented with 50 μg/mL ampicillin at 30°C till to stationary stage. The total amount of RNA was extracted for relative expression analysis of the genes, including *smpB*, the predicted targets of AvrA (t15190, t21425, t18380, t22295, t14250), Fe/S cluster assembly regulation factor (*iscR*) and its regulated targets *iscA, iscU, iscS*.

### Florescent Measurement

The cells conferring eGFP expression were suspended in PBS buffer, and loaded to a 96 well microtiter plate (Greiner BIOONE, Nurnberg, Germany). The fluorescent intensity was measured at 488 nm excitation and 540 nm emission wavelengths with a fluorescence microplate reader (Infinite® 200 PRO, Tecan, Shanghai, China). The relative fluorescence was calculated as the total fluorescence divided by OD_600_ value.

### Statistical Analysis

Statistical data were analyzed using the statistical Package for the Social Science (SPSS) version 20.0 (SPSS, Chicago, IL, United States) and GraphPad Prism version 6.0 (GraphPad, San Diego, CA, United States). The results were presented as mean values of three independent experiments with standard deviation (SD) using one-way analysis of variance (ANOVA). *P* < 0.05 or 0.01 were represented as significant or extremely significant.

## Results

### Transcriptomic Analysis

**Figure 1:**
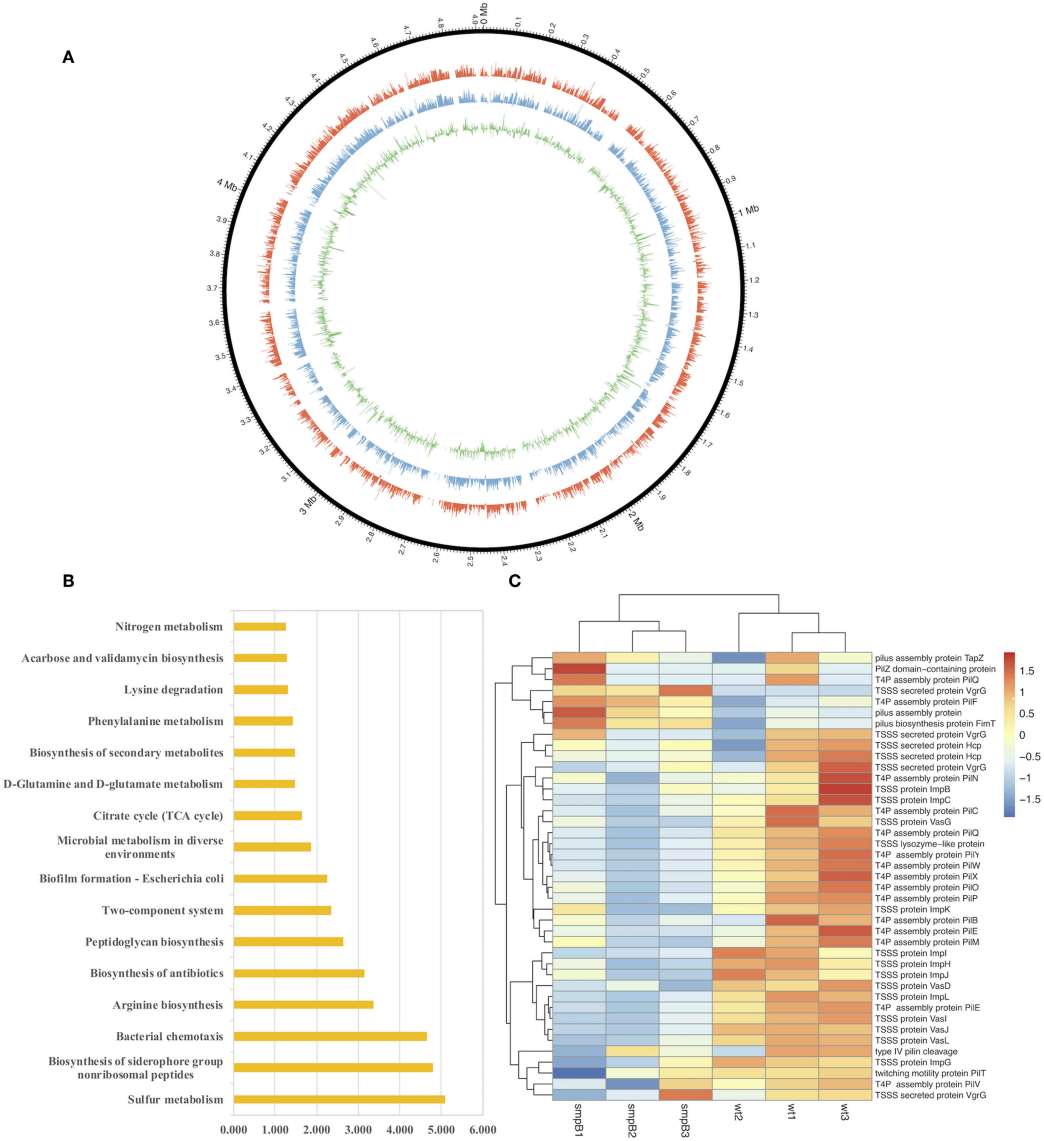
Transcriptomic analysis between wild type and s*mpB* knockout. **(A)** Circos diagram of read distribution and gene expression. From inside, green (fold change of gene expression between Δ*smpB* and wild type), blue (mRNA expression of wild type *A. veronii* by rpkm), red (mRNA expression of Δ*smpB* by rpkm), black (reads distribution on the reference genome NZ_CP012504.1). **(B)** Pathways affected by *smpB*. The horizontal and vertical axes represented as –log(*p*) value and the pathway names (*p* < 0.05). **(C)** Heat map representing the expressions of type IV pilus assembly, type VI secretion system and outer membrane genes.

To determine the differences in pathways and gene expressions between wild type and *smpB* knockout (Δ*smpB*) in the M9 medium, the transcriptome assembly of *A. veronii* was generated from 91.17 million paired-end RNA-seq reads using Illumina Xten technology and a total of 22,055 transcripts were mapped by genome NZ_CP012504.1 (Figure 1A). Sixteen pathways were significantly affected by SmpB knockout, including sulfur metabolism, biosynthesis of siderophore group non-ribosomal peptides, bacterial chemotaxis, arginine and peptidoglycan biosynthesis, and microbial metabolism in a diverse environment (Figure 1B). Among them, 21 and 20 genes were downregulated, respectively in the type IV pilus assembly and the type VI secretion system at the stationary phase (Figure 1C). The type IV pili are ubiquitously expressed on the surface of many Gram-negative bacteria and are important virulence factors that facilitate host-pathogen interactions and persistence of infection. T6SS is the molecular machine, which transports the virulence effectors from the interior cytoplasm or cytosol into an adjacent target cell across the cellular envelope. The downregulated pilus and secretion system proteins revealed that toxicity might be attenuated in Δ*smpB*.

### SmpB Deletion Decreases the Colonization in Host Tissue

**Figure 2:**
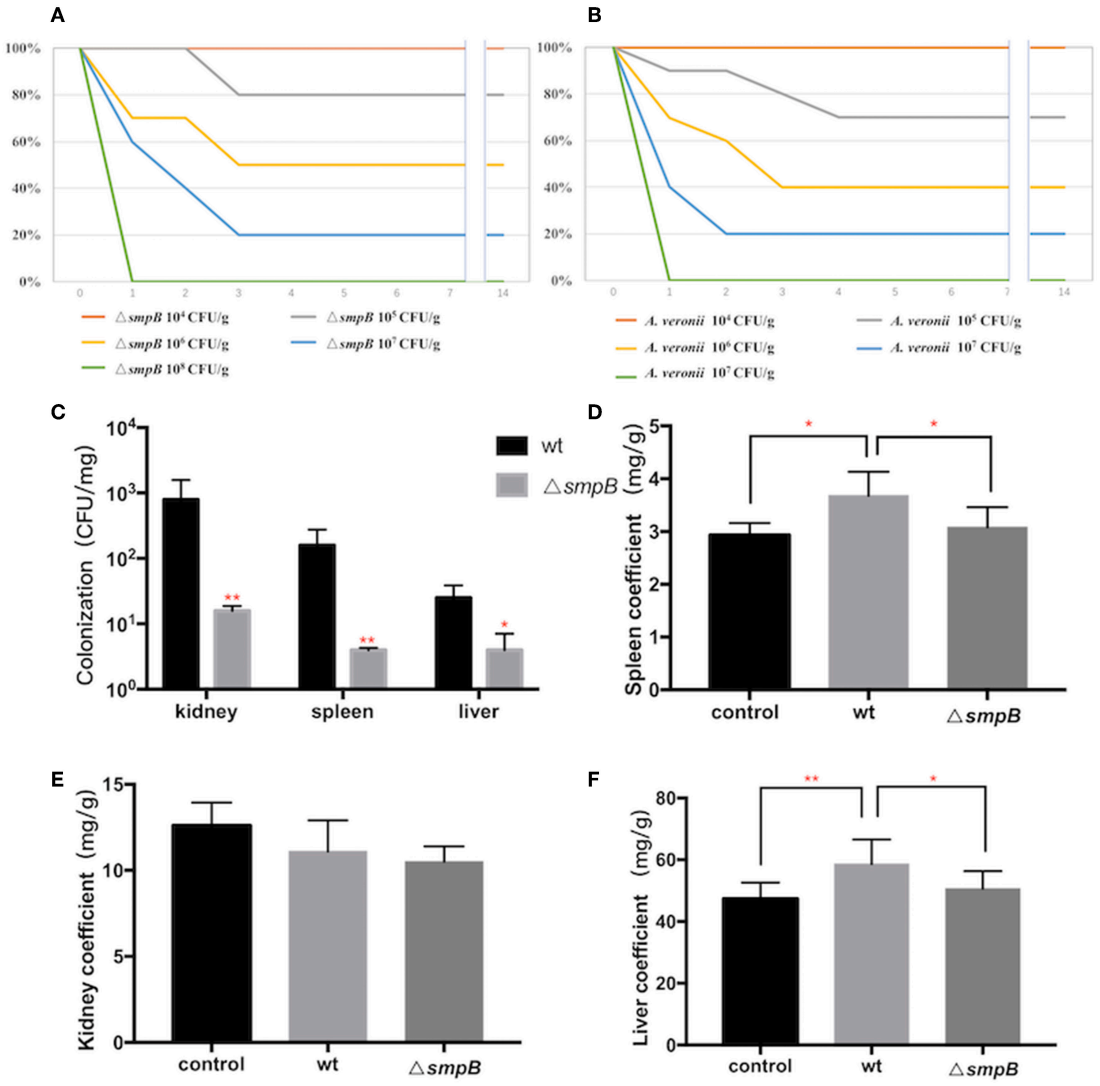
SmpB deletion decreases the colonization in host tissue. **(A)** Survival rate of mice treated by wild type *A. veronii*. **(B)** Survival rate of mice treated by Δ*smpB* strain. **(C)** Colonization of wild type and Δ*smpB* in spleen, kidney, and liver of ICR mice. **(D)** Spleen coefficient (mg/g). **(E)** Kidney coefficient (mg/g). **(F)** Liver coefficient (mg/g). Error bars were shown as SD. ^**^p < 0.01, ^*^p < 0.05, following by one-way ANOVA, independent *t*-tests.

To compare the virulence between wild type and Δ*smpB*, Institute of Cancer Research (ICR) mice were divided into 10 groups with 10^4^–10^8^ CFU/g bacterial infection. The survival rate of ICR mice revealed that the virulence of the wild type was no different compared with the *smpB* deletion (Figures 2A,B).

After the mice were intraperitoneally injected with 5 × 10^5^ CFU/g bacterial strains, the liver, spleen and kidneys were collected, and tissue suspension was cultured on LB plates supplemented with ampicillin. The wild type was colonized significantly more than Δ*smpB* in the kidney, liver, and spleen (Figure 2C), and the results agreed with the downregulated expressions of the type IV pilus assembly proteins (Figure 1C). Organ coefficient is a common toxicology indicator of ratio of organ and body weight reflecting animal damage (Zhang et al., 2014). In our study, the coefficients of spleen and liver infected by the wild type were 3.68 ± 0.45 and 58.65 ± 7.94 mg/g, which were significantly higher than the control group (2.93 ± 0.23 and 47.38 ± 5.27 mg/g) and Δ*smpB* group (3.08 ± 0.38 and 50.65 ± 5.62 mg/g) (Figures 2D,E), while little difference was observed in the kidney (Figure 2E). Next the organ coefficient was evaluated for the visceral injury between the treatment and the control group. The coefficients of the spleen and liver infected by the wild type were significantly higher than the control group and Δ*smpB* group (Figures 2D–F), while little difference was observed in the kidney (Figure 2E). This also suggested that the virulence of wild type *A. veronii* was not affected by *smpB* deletion. Consistently, the acute LD50 for Δ*smpB* strain in mice was calculated as 5 × 10^5^ CFU/g, which was close to that of 10^6^ CFU/g for the wild type. Because of the contradiction between the decreased pathogenicity and unvaried lethality, we speculated that the bacterial resistance had enhanced in the host.

### The Combination of the Downregulated AvrA and Upregulated IscR Mediates the Oxidative Tolerance in smpB Deletion

**Figure 3:**
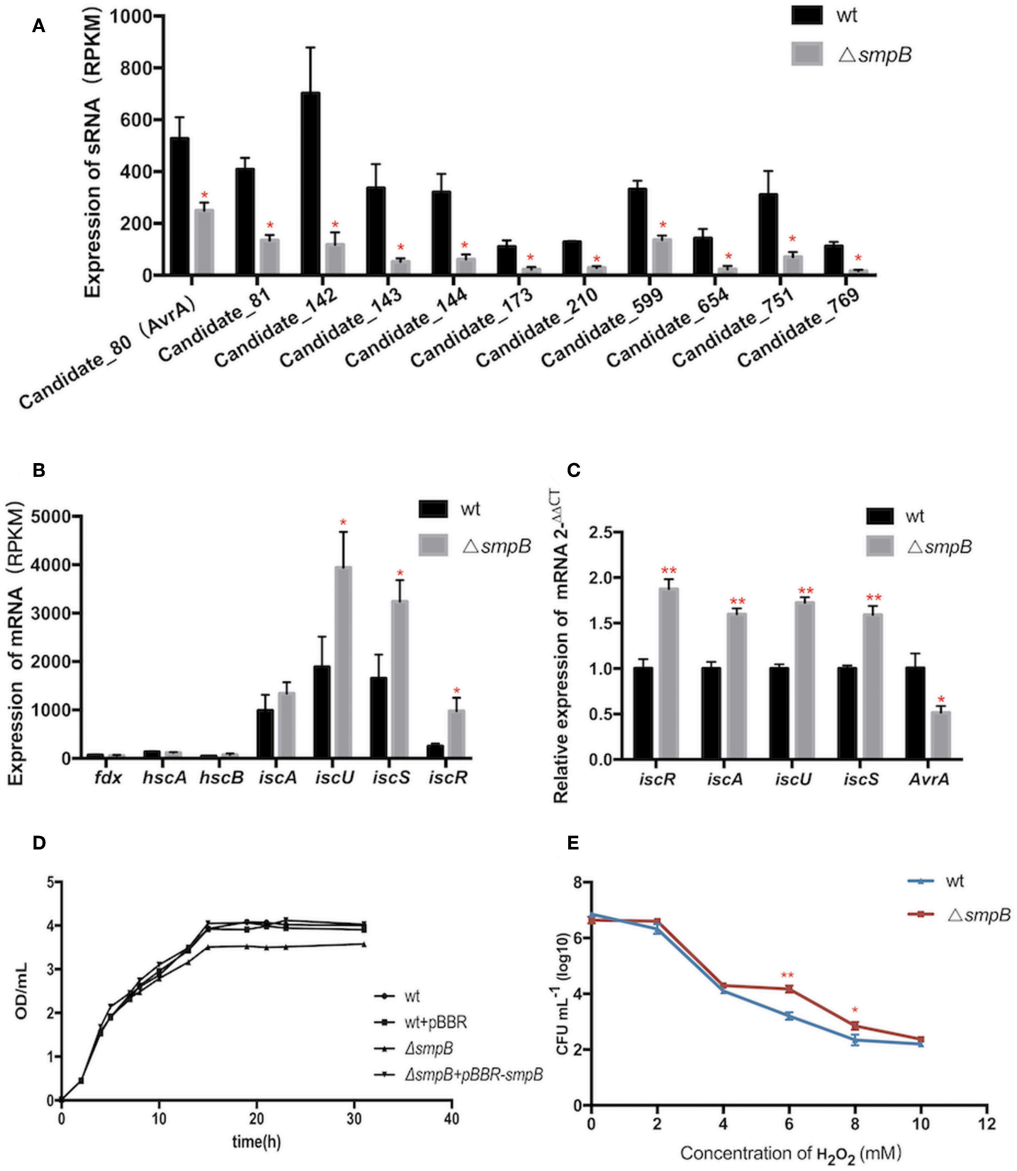
The AvrA downregulation and IscR upregulation mediate the oxidative tolerance of Δ*smpB*. **(A)** Transcriptomic comparisons of 11 sRNA between wild type and Δ*smpB*. **(B)** Transcriptomic comparisons of *iscR* operon genes between wild type and Δ*smpB*. **(C)** Realtime qPCR detection of *iscR* operon and AvrA. **(D)** The growth curves of the derivative *A. veronii*. **(E)** The survivals of Δ*smpB* and wild type in H_2_O_2_ concentrations ranging from 0 to 10 mM. ^**^p < 0.01, ^*^p < 0.05, following by one-way ANOVA, independent *t*-tests.

Expressions of transcripts were compared between the wild type and *smpB* mutant in the transcriptomic data. A total of 466 non-coding transcripts were defined as sRNA, including 50–500 nt. Among them, 11 sRNAs were analyzed to express specifically between the wild type and Δ*smpB* at the stationary stage in the M9 medium (Figure 3A). There were 62 differentially expressed target genes of the 11 sRNAs involved in stress response pathways, pathogenesis, and also the regulations of metabolism, transport, quorum sensing (Supplementary Table 1). The pathways were associated with the synthesis of cell membrane, protein translocation, ATPase, Fe/S protein assembly and energy metabolism (Table 3). The candidate_80 sRNA, designated as AvrA (*Aeromonas veronii* non-coding RNA A) was potentially associated with the target gene *iscR* in Fe/S assembly. After *smpB* was knocked out, AvrA expression declined (Figure 3A) resulting in the increased expression of the predicted targets *isc* operon genes which encoded iron-sulfur proteins (Figure 3B). The similar tendency of AvrA and its target genes was also turned out by Real-time PCR (Figure 3C).

**Table 3:** sRNAs and Pathways effected in Δ*smpB*.

**Candidate sRNA**	**Metabolic pathways Involved**
Candidate_80(AvrA)	Iron-sulfur cluster assembly
Candidate_81	Cellular homeostasis
Candidate_142	Ubiquitylation process
Candidate_143	Coenzyme biosynthetic process
Candidate_144	Intrinsic component of membrane
Candidate_173	Amino acid metabolism
Candidate_210	Purine ribonucleotide catabolic process
Candidate_599	Wide pore channel activity
Candidate_654	Energy metabolism
Candidate_751	Energy metabolism
Candidate_769	Aerobic respiration

To evaluate whether *smpB* mutation lived better in stress conditions than the wild type, the iron deficiency condition was performed to mimic the host environment and the tolerance to hydrogen peroxide oxidation (H_2_O_2_) was tested. The *smpB* mutation exhibited a slow growth compared with the wild type and the complementary strain (Figure 3D). The Δ*smpB* survived better than the wild type when the H_2_O_2_ concentration ranged from 6 to 10 mM, revealing that Δ*smpB* was endowed with a stronger capability of growth under adverse conditions (Figure 3E).

### AvrA Deletion Increases the Tolerances to Iron Deficiency and Oxidative Stress by Upregulating Iron-Sulfur Gene Expression

**Figure 4:**
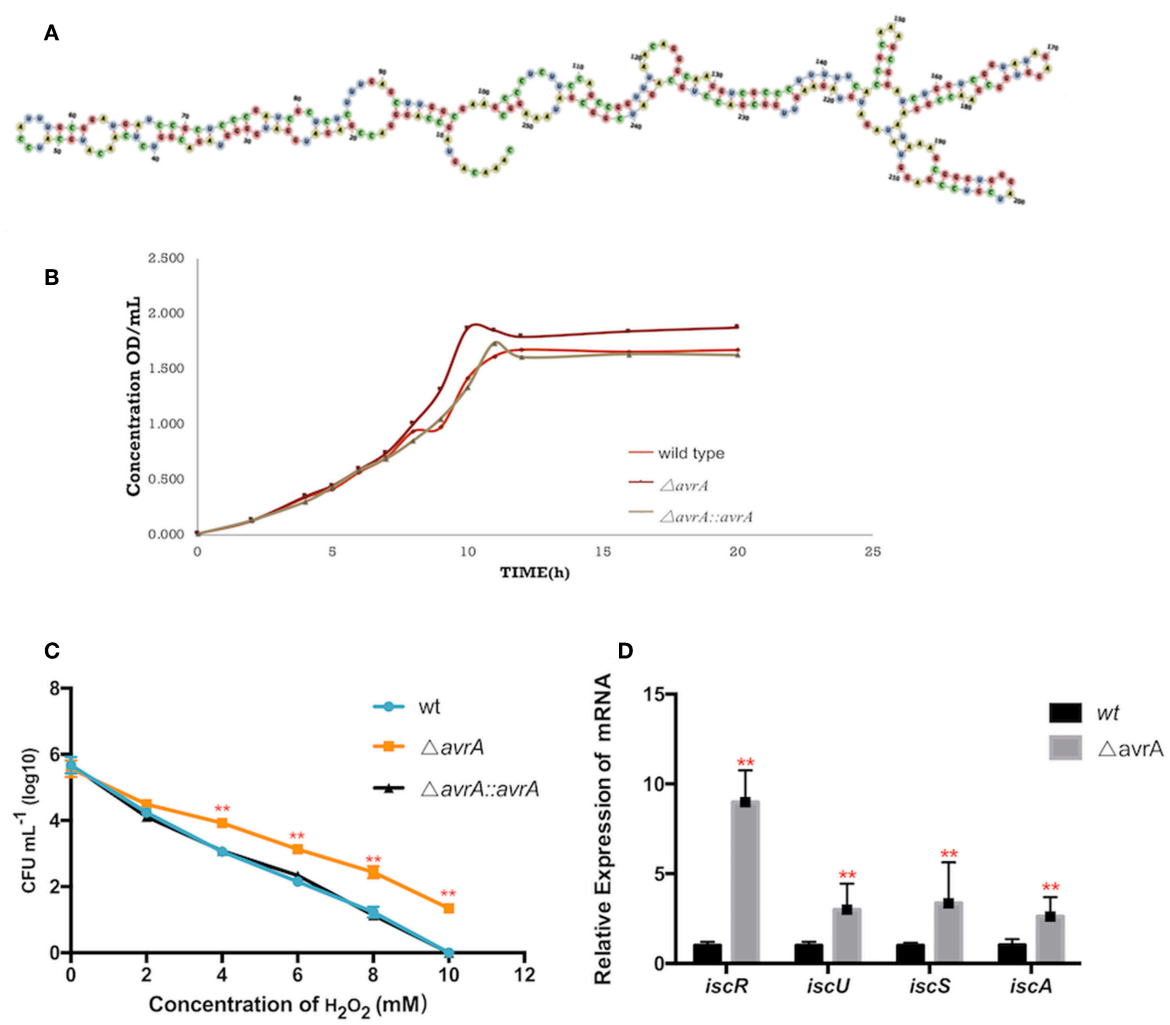
AvrA increases the tolerances to oxidative stress and iron deficiency. **(A)** The secondary structure prediction of AvrA by software RNAfold. **(B)** Growth curves of wild type, Δ*avrA* and the complemented strain in iron deficiency treatment. **(C)** The survivals of wild type, Δ*avrA* and the complemented strain under H_2_O_2_ stress in the concentrations ranging from 0 to 10 mM **(D)** Relative expression of *iscR* operon at stationary phage in iron deficiency treating with DIP. ^**^p < 0.01, following by one-way ANOVA, independent *t*-tests.

AvrA was a non-coding RNA of 253nt, and its secondary structure was predicted by the software RNAfold (http://rna.tbi.univie.ac.at/cgi-bin/RNAWebSuite/RNAfold.cgi) (Figure 4A). The growth results demonstrated that the AvrA deletion presented a better ability to grow under dipyridyl (DIP)-chelated iron deficiency state (Figure 4B). Also, the oxidation resistance of Δ*avrA* was evaluated under 0–10 mM H_2_O_2_ in comparison with the wild type and the complemented strains, showing that the antioxidation capacity was enhanced after AvrA knockout (Figure 4C). Realtime qPCR results showed that the transcriptional expressions of the iron-sulfur genes *iscR, iscU, iscS, and iscA* were significantly increased (Figure 4D).

### AvrA Binds and Downregulates iscR mRNA by Base Pairing

**Figure 5:**
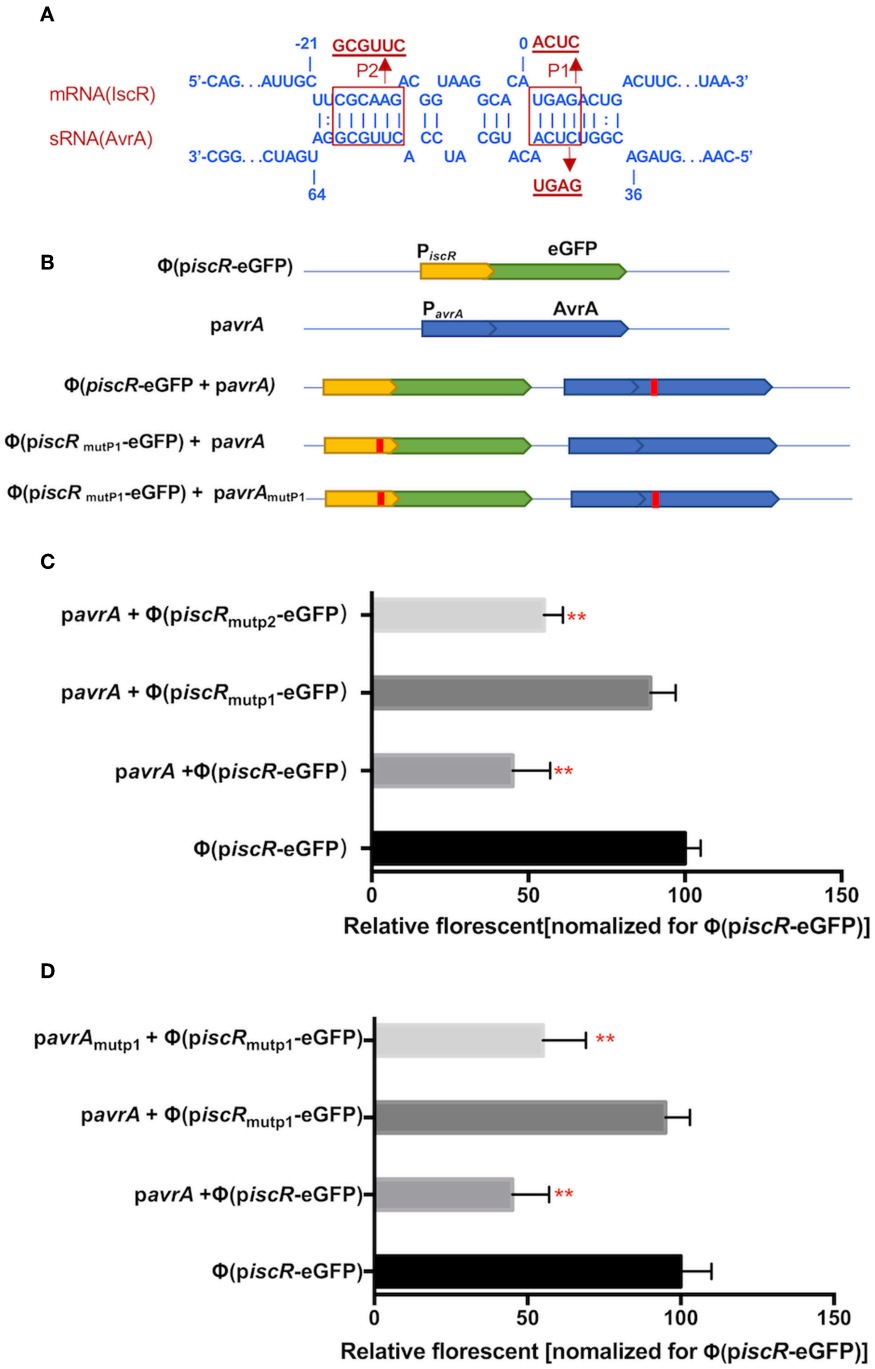
AvrA interacts with *iscR* mRNA by base pairing. **(A)** Predicted interaction between AvrA and *iscR* mRNA by the RNAplex program. **(B)** Basic architecture of the plasmid constructs. **(C,D)** The relative florescence was measured at 488 nm excitation and 520 nm emission lights after the corresponding constructs were co-transformed in *E. coli* strain. ^**^p < 0.01, following by one-way ANOVA, independent *t*-tests.

The potential interaction site was predicted between AvrA and the *iscR* promotor using the RNAplex program. Interestingly, the base-pairing region encompassed the ribosome binding site and the start codon AUG of *iscR*, and the binding site spanned from C_37_ to A_63_ of 5′-end AvrA (Figure 5A). Two mutations, P1 (from A_2_ to C_5_, ACUC) and P2 (from C_−18_ to G_−13_, GCGUUC), were introduced in the *iscR* promoter (*piscR*) and fused with the enhanced green fluorescent protein (eGFP) to produce Φ(p*iscR*_mutP1_-eGFP) and Φ(p*iscR*_mutP2_-eGFP), respectively (Figures 5A,B). When AvrA was co-expressed with Φ(p*iscR*-eGFP), the fluorescence was repressed, suggesting that AvrA could interact with the promotor of *iscR* by base pairing (Figure 5C). Furthermore, the fluorescence recovered when AvrA was co-transformed with Φ(p*iscR*_mutP1_-eGFP), revealing that P1 was the exact site for interaction (Figure 5C). Next, a mutation named p*avrA*_mutP1_ was introduced in the AvrA expression plasmid p*avrA* to match Φ (p*iscR*_mutP1_-eGFP), resulting in fluorescent repression. Conversely the mutated p*avrA*_mutP1_ did not reduce the fluorescence when co-expressed with the wild type Φ(*iscR*-eGFP) (Figure 5D). The results concluded that AvrA repressed the expression of *iscR* by base pairing with the *iscR* translation initiation region P1.

## Discussion

SmpB is not only the key component of the trans-translation system, but also plays an important role as a transcription regulator in the global regulation of bacteria (Liu et al., 2016). In our study, the *smpB* deletion affected the transcriptional expression of the genes related to the bacterial growth, metabolism and protein secretion in multiple metabolic pathways in *A. veronii*. Previously Type II, III, and VI secretion systems were reported in multiple strains of *Aeromonas* bacteria, which function by exporting bacterial virulence proteins and by infecting host cells (Suarez et al., 2009; Journet and Cascales, 2016). The gene expression of the Type III secretion system had extremely low abundance, while those of Type VI performed higher in the transcriptome. Most of all, virulence-related genes which mediated the pili assembly (type IV pilus) and protein secretion (type VI secretion system, TSSS) were totally downregulated (Figure 1). Previously, the type VI secretion system was identified to function as the virulence factor in *Vibrio hollisae* and *Aeromonas hydrophila* (Russell et al., 2014). The colonization of wild type *A. veronii* was significantly higher than that of Δ*smpB*. However, the survival rates of the infected mice showed no significant differences between the wild type and Δ*smpB* strain. Previously the *smpB* deletion mutant of *Listeria monocytogenes* and *Salmonella Typhimurium* were avirulent and deficient in the intramacrophage survival (Kivisaar, 2003; Mraheil et al., 2017) The results were inconsistent with past reports, indicating that there are other pathways maintaining the virulence in *A. veronii smpB* mutants. Subsequently, sRNA AvrA was revealed as an alternative for virulence compensation. The downregulation of AvrA affected the expression of IscR in Δ*smpB A. veronii*. In case of iron deficiency, IscR enhanced iron-sulfur cluster assembly and iron sulfur protein synthesis (Santos et al., 2014), and amended the antioxidant ability of *A. veronii*.

**Figure 6:**
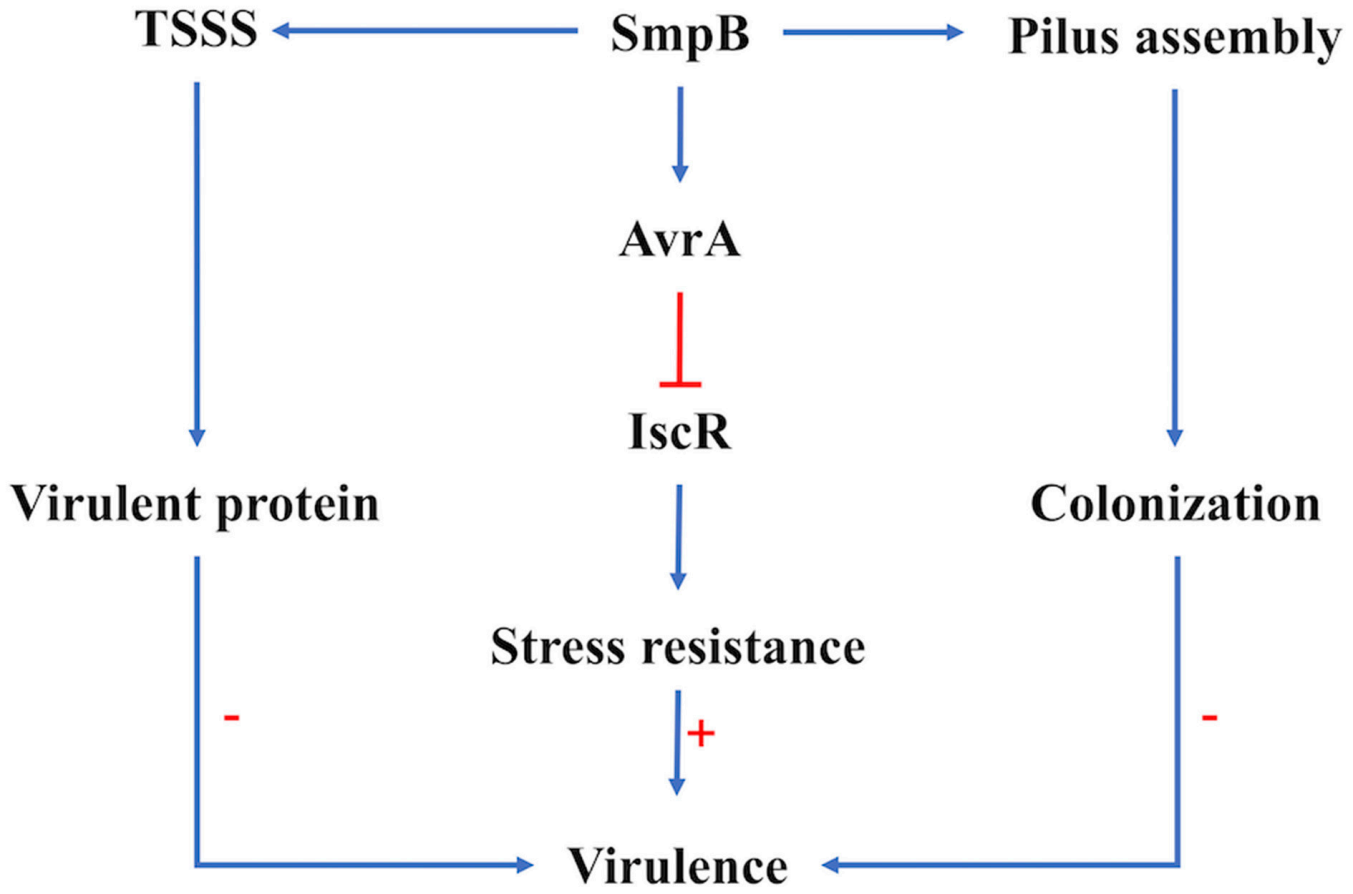
Regulation of SmpB in virulence regulation. The virulence of *A. veronii* was affected by the expression of virulent proteins the ability of host colonization and the resistance to adversity. Type VI secretion system and pilus assembly were affected on the transcription and translation level by SmpB regulation directly. The sRNA AvrA functioned the stress resistance directly, which was mediated by SmpB indirectly. The arrows in blue showed the positive regulation, and the line in red showed repression. The symbol “+”, “–” indicated upregulation and downregulation, respectively.

When bacteria infect the host, they are subjected to stressful conditions such as strong oxidization, deficient nutrition and metal ion. The harsh conditions cause starvation, accumulation of secondary metabolites, decreased fidelity of DNA replication and the reduction in DNA repair activity which are similarly encountered during the stationary stage (De Biase et al., 1999; Kivisaar, 2003). Most sRNAs are expressed as important regulators in the process of gene regulation under adverse stress (Michaux et al., 2014; Amin et al., 2016; Holmqvist and Wagner, 2017). The sRNA AvrA was uncovered to function by base pairing with its target gene *iscR*. IscR regulated a set of genes involved in iron-sulfur cluster assembly in adversity like iron deficiency and oxidative stress (Figure 6). However, the transcription factors associated with oxidative stress, including Fur (ferric uptake regulation protein), OxyR (hydrogen peroxide-inducible genes activator), SoxR (hydrogen peroxide-inducible genes activator) were insignificantly expressed by comparing Δ*smpB* strains the with wild type strains (Supplementary Table 2). The mutation of *smpB* resulted in the downregulation of sRNA AvrA, which leads to the upregulation of *iscR* expression and the enhancement of the adverse resistance. Combined with the attenuated virulence and the increased survival in adversity in Δ*smpB*, the strains performed a similar lethality in mice compared with the wild type. Virulence is a trait that has been selected in evolutionary history (Rafaluk et al., 2015; Gerstein and Nielsen, 2017). Therefore, the survival viability in the macrophage and the tolerance to adverse environments caused by *smpB* mutation compensates for the loss of function in virulence, causing *A. veronii* to evolve as a superbug.

## Data Availability

The raw data supporting the conclusions of this manuscript will be made available by the authors, without undue reservation, to any qualified researcher.

## Author Contributions

ZL, XM, and DW contributed the conception and design of the study. DW, HL, YT, HT, and ZL performed the statistical analysis. DW, HL, XH, and ZL drafted the manuscript. All authors contributed to manuscript revision, read and approved the submitted version.

### Conflict of Interest Statement

The authors declare that the research was conducted in the absence of any commercial or financial relationships that could be construed as a potential conflict of interest.

## References

[B1] Aguilera-Arreola M. G. Hernandez-Rodriguez C. Zuniga G. Figueras M. J. Garduno R. A. Castro-Escarpulli G. (2007). Virulence potential and genetic diversity of *Aeromonas caviae, Aeromonas veronii*, and *Aeromonas hydrophila* clinical isolates from Mexico and Spain: a comparative study. Can. J. Microbiol. 53, 877–887. 10.1139/W07-051 17898843

[B2] Amin S. V. Roberts J. T. Patterson D. G. Coley A. B. Allred J. A. Denner J. M.. (2016). Novel small RNA (sRNA) landscape of the starvation-stress response transcriptome of *Salmonella enterica* serovar typhimurium. RNA Biol. 13, 331–342. 10.1080/15476286.2016.1144010 26853797 PMC4829330

[B3] Carrier M.-C. Bourassa J.-S. Massé E. (2017). Cellular homeostasis: a small RNA at the crossroads of iron and photosynthesis. Curr. Biol. 27, R380–R383. 10.1016/j.cub.2017.04.003 28535387

[B4] De Biase D. Tramonti A. Bossa F. Visca P. (1999). The response to stationary-phase stress conditions in *Escherichia coli* : role and regulation of the glutamic acid decarboxylase system. Mol. Microbiol. 32, 1198–1211. 10383761 10.1046/j.1365-2958.1999.01430.x

[B5] Durand S. Tomasini A. Braun F. Condon C. Romby P. (2015). sRNA and mRNA turnover in gram-positive bacteria. FEMS Microbiol. Rev. 39, 316–330. 10.1093/femsre/fuv007 25934118

[B6] Fiedler M. R. M. Cairns T. C. Koch O. Kubisch C. Meyer V. (2018). Conditional expression of the small GTPase ArfA impacts secretion, morphology, growth, and actin ring position in *Aspergillus niger*. Front. Microbiol. 9:878. 10.3389/fmicb.2018.00878 29867795 PMC5952172

[B7] Gerstein A. C. Nielsen K. (2017). It's not all about us: evolution and maintenance of *Cryptococcus* virulence requires selection outside the human host. Yeast 34, 143–154. 10.1002/yea.3222 27862271 PMC7528103

[B8] Haines S. Arnaud-Barbe N. Poncet D. Reverchon S. Wawrzyniak J. Nasser W.. (2015). IscR regulates synthesis of colonization factor antigen I fimbriae in response to iron starvation in enterotoxigenic *Escherichia coli*. J. Bacteriol. 197, 2896–2907. 10.1128/JB.00214-15 26124243 PMC4542172

[B9] Himeno H. Kurita D. Muto A. (2014). tmRNA-mediated trans-translation as the major ribosome rescue system in a bacterial cell. Front. Genet. 5:66. 10.3389/fgene.2014.00066 24778639 PMC3985003

[B10] Holmqvist E. Wagner E. G. H. (2017). Impact of bacterial sRNAs in stress responses. Biochem. Soc. Trans. 45, 1203–1212. 10.1042/BST20160363 29101308 PMC5730939

[B11] Huter P. Muller C. Arenz S. Beckert B. Wilson D. N. (2017). Structural basis for ribosome rescue in bacteria. Trends Biochem. Sci. 42, 669–680. 10.1016/j.tibs.2017.05.009 28629612

[B12] Ilbert M. Bonnefoy V. (2013). Insight into the evolution of the iron oxidation pathways. Biochim. Biophys. Acta 1827, 161–175. 10.1016/j.bbabio.2012.10.001 23044392

[B13] Journet L. Cascales E. (2016). The type VI secretion system in *Escherichia coli* and related species. EcoSal Plus 7:20. 10.1128/ecosalplus.ESP-0009-2015 27223818 PMC11575709

[B14] Keiler K. C. (2008). Biology of trans-translation. Annu. Rev. Microbiol. 62, 133–151. 10.1146/annurev.micro.62.081307.162948 18557701

[B15] Keiler K. C. (2015). Mechanisms of ribosome rescue in bacteria. Nat. Rev. Microbiol. 13, 285–297. 10.1038/nrmicro3438 25874843

[B16] Kivisaar M. (2003). Stationary phase mutagenesis: mechanisms that accelerate adaptation of microbial populations under environmental stress. Environ. Microbiol. 5, 814–827. 10.1046/j.1462-2920.2003.00488.x 14510835

[B17] Kurita D. Miller M. R. Muto A. Buskirk A. R. Himeno H. (2014). Rejection of tmRNA·SmpB after GTP hydrolysis by EF-Tu on ribosomes stalled on intact mRNA. RNA 20, 1706–1714. 10.1261/rna.045773.114 25246654 PMC4201823

[B18] Li J. Ji L. Shi W. Xie J. Zhang Y. (2013). Trans-translation mediates tolerance to multiple antibiotics and stresses in *Escherichia coli*. J. Antimicrobial Chemother. 68, 2477–2481. 10.1093/jac/dkt231 23812681 PMC3797643

[B19] Liang W. Deutscher M. P. (2010). A novel mechanism for ribonuclease regulation: transfer-messenger RNA (tmRNA) and its associated protein SmpB regulate the stability of RNase R. J. Biol. Chem. 285, 29054–29058. 10.1074/jbc.C110.168641 20688916 PMC2937936

[B20] Liu P. Chen Y. Wang D. Tang Y. Tang H. Song H.. (2016). Genetic selection of peptide aptamers that interact and inhibit both small protein B and alternative ribosome-rescue factor A of *Aeromonas veronii* C4. Front. Microbiol. 7:1228. 10.3389/fmicb.2016.01228 27588015 PMC4988972

[B21] Liu Z. Hu K. Tang Y. Li H. Tang H. Hu X.. (2018). SmpB down-regulates proton-motive force for the persister tolerance to aminoglycosides in *Aeromonas veronii*. Biochem. Biophys. Res. Commun. 507, 407–413. 10.1016/j.bbrc.2018.11.052 30449596

[B22] Liu Z. Liu P. Liu S. Song H. Tang H. Hu X. (2015). Small protein B upregulates sensor kinase bvgS expression in *Aeromonas veronii*. Front. Microbiol. 6:579. 10.3389/fmicb.2015.00579 26136727 PMC4468919

[B23] Mandin P. Chareyre S. Barras F. (2016). A regulatory circuit composed of a transcription factor, IscR, and a regulatory RNA, RyhB, controls Fe-S cluster delivery. mBio 7:e00966-16. 10.1128/mBio.00966-16 27651365 PMC5040110

[B24] Michaux C. Verneuil N. Hartke A. Giard J.-C. (2014). Physiological roles of small RNA molecules. Microbiology 160(Pt. 6), 1007–1019. 10.1099/mic.0.076208-0 24694375

[B25] Mraheil M. A. Frantz R. Teubner L. Wendt H. Linne U. Wingerath J.. (2017). Requirement of the RNA-binding protein SmpB during intracellular growth of *Listeria monocytogenes*. Int. J. Med. Microbiol. 307, 166–173. 10.1016/j.ijmm.2017.01.007 28202229

[B26] Mu X. Huan H. Xu H. Gao Q. Xiong L. Gao R.. (2013). The transfer-messenger RNA-small protein B system plays a role in avian pathogenic *Escherichia coli* pathogenicity. J. Bacteriol. 195, 5064–5071. 10.1128/JB.00628-13 24013628 PMC3811600

[B27] Oliva G. Sahr T. Buchrieser C. (2015). Small RNAs, 5′ UTR elements and RNA-binding proteins in intracellular bacteria: impact on metabolism and virulence. FEMS Microbiol. Rev. 39, 331–349. 10.1093/femsre/fuv022 26009640

[B28] Rafaluk C. Gildenhard M. Mitschke A. Telschow A. Schulenburg H. Joop G. (2015). Rapid evolution of virulence leading to host extinction under host-parasite coevolution. BMC Evol. Biol. 15:112. 10.1186/s12862-015-0407-0 26070343 PMC4464865

[B29] Roche B. Aussel L. Ezraty B. Mandin P. Py B. Barras F. (2013). Iron/sulfur proteins biogenesis in prokaryotes: formation, regulation and diversity. Biochim. Biophys. Acta 1827, 455–469. 10.1016/j.bbabio.2012.12.010 23298813

[B30] Russell A. B. Wexler A. G. Harding B. N. Whitney J. C. Bohn A. J. Goo Y. A.. (2014). A type VI secretion-related pathway in bacteroidetes mediates interbacterial antagonism. Cell Host Microbe 16, 227–236. 10.1016/j.chom.2014.07.007 25070807 PMC4136423

[B31] Santos J. A. Alonso-Garcia N. Macedo-Ribeiro S. Pereira P. J. (2014). The unique regulation of iron-sulfur cluster biogenesis in a Gram-positive bacterium. Proc. Natl. Acad. Sci. U S A. 111, E2251–E2260. 10.1073/pnas.1322728111 24847070 PMC4050560

[B32] Suarez G. Sierra J. C. Erova T. E. Sha J. Horneman A. J. Chopra A. K. (2009). A type VI secretion system effector protein, VgrG1, from *Aeromonas hydrophila* that induces host cell toxicity by ADP ribosylation of actin. J. Bacteriol. 192, 155–168. 10.1128/jb.01260-09 19880608 PMC2798274

[B33] Torii Y. Goto Y. Nakahira S. Kozaki S. Kaji R. Ginnaga A. (2015). Comparison of Systemic toxicity between botulinum toxin subtypes A1 and A2 in mice and rats. Basic Clin. Pharmacol. Toxicol. 116, 524–528. 10.1111/bcpt.12351 25395371

[B34] Xu X. M. Moller S. G. (2008). Iron-sulfur cluster biogenesis systems and their crosstalk. Chembiochem 9, 2355–2362. 10.1002/cbic.200800384 18798211

[B35] Zhang R. Zhang L. Jiang D. Zheng K. Cui Y. Li M.. (2014). Mouse organ coefficient and abnormal sperm rate analysis with exposure to tap water and source water in Nanjing reach of Yangtze River. Ecotoxicology 23, 641–646. 10.1007/s10646-014-1228-4 24664459

